# Increased fluorescence observation intensity during the photodynamic diagnosis of deeply located tumors by fluorescence photoswitching of protoporphyrin IX

**DOI:** 10.1117/1.JBO.28.5.055001

**Published:** 2023-05-15

**Authors:** Sochi J. Ogbonna, William Y. York, Takahiro Nishimura, Hisanao Hazama, Hideo Fukuhara, Keiji Inoue, Kunio Awazu

**Affiliations:** a Osaka University, Graduate School of Engineering, Division of Sustainable Energy and Environmental Engineering, Osaka, Japan; b National Institute on Aging, Laboratory of Clinical Investigation, Baltimore, Maryland, United States; c Kochi University, Kochi Medical School, Department of Urology, Kochi, Japan; d Osaka University, Global Center for Medical Engineering and Informatics, Osaka, Japan

**Keywords:** fluorescence photoswitching, photodynamic diagnosis, 5-aminolevulinic acid, protoporphyrin IX, photobleaching, photoproduct

## Abstract

**Significance:**

Photobleaching of the photosensitizer reduces fluorescence observation time and the intensity of fluorescence emitted for tumor detection during 5-aminolevulinic acid-based photodynamic diagnosis.

**Aim:**

This study aims to utilize the concept of fluorescence photoswitching, which uses the fluorescence emission from photosensitizer excitation followed by the simultaneous excitation of the photosensitizer and its photoproduct to increase the fluorescence detection intensity during PDD of deeply located tumors.

**Approach:**

The fluorescence photobleaching of protoporphyrin IX (PpIX) and the formation of its photoproduct, photoprotoporhyrin (Ppp), caused by exposure to 505 nm light were investigated in solution, *ex vivo*, and *in vivo*, and the fluorescence photoswitching was analyzed. The fluorescence observations of PpIX and Ppp were performed with 505 and 450 or 455 nm excitation, respectively, which is the suited wavelength for the primary excitation of each fluorophore.

**Results:**

Fluorescence photoswitching was observed in all forms of PpIX investigated, and the fluorescence photoswitching time, fluorescence intensity relative to the initial PpIX and Ppp intensity, and fluorescence intensity relative to PpIX after photobleaching were obtained. The dependence of the fluorescence photoswitching time and intensity on the irradiation power density was noted. A fluorescence intensity increase between 1.6 and 3.9 times was achieved with simultaneous excitation of PpIX and Ppp after fluorescence photoswitching, compared with the excitation of PpIX alone.

**Conclusions:**

We have demonstrated the potential of fluorescence photoswitching for the improvement of the fluorescence observation intensity for the PDD of deeply located tumors.

## 1 Introduction

Photodynamic diagnosis (PDD) using 5-aminolevulinic acid-induced protoporphyrin IX (ALA-induced PpIX) for the visualization and detection of various cancers is widespread.^1^^–^^4^ PpIX, used as the photosensitizer in this technique, is a natural intermediate in the heme synthesis pathway and is generated from its precursor ALA. PpIX is characterized by its selective accumulation in cancer cells, and it is fluorescent when excited at an appropriate wavelength, leading to cancer cell detection.^5^^–^^7^ Although this diagnostic technique is minimally invasive and offers high specificity for cancer cell detection,^1^^,^^2^^,^^8^ the diagnostic efficacy of PDD is affected by some factors, including the wavelength of the excitation light and the photobleaching of PpIX.

Different wavelength ranges can be used in the excitation of PpIX to induce fluorescence for tumor detection. However, their penetration depths in tissue vary due to the attenuation of light via absorption and scattering in tissue.^9^^,^^10^ Thus, the efficiency of the photosensitizer excitation, and, consequently, the diagnostic depth, is affected by the excitation wavelength, tissue optical properties, and tumor location. Increased tissue penetration and reduced scattering of the excitation light can be achieved with longer wavelengths.^9^^,^^11^ Investigations on the use of red light excitation around 633 to 635 nm for fluorescence detection confirmed increased penetration depth of the excitation light.^10^^,^^12^ However, tumor detection with red light excitation of PpIX might be challenging in clinical applications, as PpIX emits red light, which overlaps with the red excitation wavelengths, resulting in minimal fluorescence detection. Various excitation wavelengths have been analyzed to maximize the fluorescence intensity of PpIX at different depths for the fluorescence detection of deeply located gastric and bladder cancer.^11^^,^^13^ Their results showed that using 505 nm light for excitation could extend the fluorescence diagnostics depth, without overlaps with the PpIX fluorescence emission. However, these studies for both red light and green light excitation of PpIX^10^^–^^13^ did not take into account the effect of photosensitizer photobleaching.

During the fluorescence diagnosis process in PDD, the photobleaching of the photosensitizer occurs. The concentration and, thus, the fluorescence intensity of the photosensitizer reduce under light exposure, decreasing the observed fluorescence.^4^^,^^14^^,^^15^ Aside from the wavelength of light used in the irradiation, the photobleaching rate of PpIX is dependent on its initial concentration.^16^^–^^18^ Goyan et al. observed a 50% reduction of 10  μM PpIX solution after 25 min of 300 mW irradiation with 764 nm light.^17^ A 90% reduction in PpIX was observed in 0.05 mM initial ALA concentration within WiDR cells after irradiation with 630 nm at $30\;\mathrm{mW}/{\mathrm{cm}}^{2}$ for $75\;J/{\mathrm{cm}}^{2}$,^16^ whereas a similar reduction of PpIX in nude mice was seen after $12\;J/{\mathrm{cm}}^{2}$ of $50\;\mathrm{mW}/{\mathrm{cm}}^{2}$ with 514 nm irradiation.^18^ Because the fluorescence emission from the photosensitizer is already low when used in detecting deeply located tumors, the effect of photobleaching is notable.

Various studies report the formation of photoproducts with PpIX photobleaching. Light irradiation of PpIX in solution, *in vitro*, and *in vivo* forms photoproducts having absorption bands in the red spectral region.^18^^–^^21^ The dominant photoproduct formed from PpIX irradiation is photoprotoporphyrin (Ppp). Ppp, in itself, is a fluorophore and emits fluorescence when excited.^22^ Hence, there is the potential use of fluorescence emission from the photoproduct for fluorescence diagnosis.

This study aims to improve the fluorescence observation of tumors located deeply, by using fluorescence photoswitching. We investigated the use of the fluorescence emission from both PpIX and its photoproduct to increase the fluorescence emission from the photosensitizer to improve the detection of deeply located tumors. Changing the excitation light to an appropriate wavelength at an appropriate time would result in fluorescence photoswitching, with photoproducts yielding fluorescence intensity higher than that of PpIX at the time. This would increase the fluorescence detection intensity and, consequently, the diagnostic efficacy. This paper investigates the fluorescence photoswitching between PpIX and its photoproduct when irradiated and excited with green and blue light at time intervals. The fluorescence intensity of PpIX and that of the photoproduct formed after PpIX photobleaching caused by 505 nm excitation were studied in solution. *Ex vivo* analyses were carried out to determine the fluorescence detection of PpIX and its photoproduct within the gastric mucosa layer under similar excitations. Analyses were also performed *in vivo* to verify the results observed in solution and *ex vivo*.

## 2 Materials and Methods

### 2.1 ALA-PDD using Fluorescence Photoswitching

**Fig. 1:**
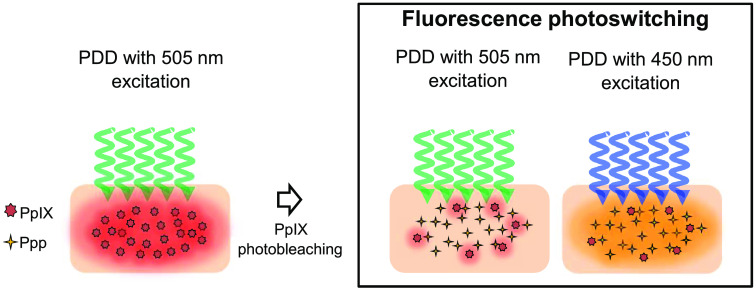
Concept of fluorescence photoswitching. The irradiation of PpIX during PDD leads to its photobleaching and the formation and accumulation of Ppp. PDD with 505 nm is primarily used to excite PpIX, whereas 450 nm is primarily used in Ppp excitation. The photobleaching of PpIX leads to a reduced fluorescence emission when excited, and the excitation of the accumulated Ppp increases fluorescence emission, leading to fluorescence photoswitching. The fluorescence emission from PpIX is shown in red, and the fluorescence emission from Ppp is shown in yellow.

The concept of fluorescence photoswitching (Fig. 1) is as follows: PDD for a deeply located tumor is performed by exciting the photosensitizer with green light. During the fluorescence detection, there is continual photobleaching of the photosensitizer, accompanied by the formation of the photosensitizer photoproducts. The photoproducts formed accumulate as the photosensitizer photobleaches. After notable photobleaching of the photosensitizer, the wavelength of the excitation light is changed to also excite the accumulated photoproducts. The fluorescence emitted from the combination of the photosensitizer and its photoproducts yield fluorescence intensity higher than that of the photobleached photosensitizer alone. Thus, the tumor detection efficacy is improved with the increased fluorescence emission intensity.

The photosensitizer used in 5-aminolevulinic acid-based photodynamic diagnosis (ALA-PDD) is PpIX. Thus, the expected dominant photoproduct is Ppp. Similar to PpIX, Ppp can be excited at various wavelengths. It has been reported that the highest relative fluorescence intensity of Ppp is observed for excitation with 450 nm.^22^ The absorption spectra of PpIX irradiated with 505 nm light (Fig. S1 in the Supplementary Material) showed a peak at 667 nm, which is absent in the unirradiated PpIX. Similar to other studies, the absorption spectra also showed the broadening of the Soret band, which increased in the absorption intensity at around 450 nm.^22^^,^^23^ A ratio of the irradiated absorption spectra to the unirradiated absorption spectra showed peaks at 520 nm in addition to the 450, 560, and 615 nm observed by Bagdonas et al.^22^ Excitation with 450 nm offers less tissue scattering and deeper penetration than 405 nm, which is commonly used in PDD.^11^ Furthermore, Ppp is less susceptible to photodegradation than PpIX;^22^^,^^24^^,^^25^ thus, there may be an extended fluorescence emission time with fluorescence photoswitching. Based on the wavelength of the excitation light, the photobleaching of Ppp differs. The Ppp photobleaching rate is slower than that of PpIX when excited at 410 nm, but a rate similar to that of PpIX, when excited at 440 nm, was reported.^26^ Another report showed that the irradiation of $8.5\;J/{\mathrm{cm}}^{2}$ at $140\;\mathrm{mW}/{\mathrm{cm}}^{2}$ was needed to halve the concentration of PpIX at 635 nm, whereas $27\;J/{\mathrm{cm}}^{2}$ was needed for a similar effect in Ppp at 670 nm.^24^

### 2.2 Sample Preparation for Spectroscopic Analysis

PpIX (P8293, Sigma-Aldrich) was diluted to 10  μM with dimethyl sulfoxide (DMSO, D4540, Sigma-Aldrich), and 60  μL of the diluted PpIX was placed per well in a clear-bottomed black 96-well plate (353219, Falcon). The samples were exposed to a green light-emitting diode (LED) with a nominal wavelength of 505 nm and a bandwidth (full-width at half-maximum; FWHM) of 30 nm (M505L3, Thorlabs). The wells were irradiated following the irradiation method described in Sec. 2.3 with an increasing energy density of 0, 10, 20, 30, 40, and $50\;J/{\mathrm{cm}}^{2}$ for power densities of 20 and $50\;\mathrm{mW}/{\mathrm{cm}}^{2}$. Each of the irradiated samples was further irradiated with an energy density of $10\;J/{\mathrm{cm}}^{2}$ using an LED with a nominal wavelength of 455 nm and a bandwidth (FWHM) of 18 nm (M455L3, Thorlabs), with the same power density used in the green-light irradiation. The irradiation conditions for the solution analysis were varied to determine the effect of irradiance on the photoproduct formation rate.

### 2.3 Irradiation

The samples in the 96-well plate, having a bottom area of $0.32\;{\mathrm{cm}}^{2}$ per well, were irradiated from the bottom, with the LED light irradiated through a collimation adapter (COP1-A, Thorlabs). The light was passed through an iris diaphragm (IH-50R-N, Sigmakoki, Japan) with a diameter set to 0.64 cm before being projected to the bottom of the well to keep the light beam diameter fixed for each well. The irradiation powers were set using a tuneable LED driver (LEDD1B, Thorlabs) and a variable neutral density filter (NDHN-50, Sigmakoki, Japan). The LED power was measured using a power sensor with a filter (PD-300 UV, Ophir Optronics, Israel) attached to a display (Nova II, Ophir Optronics). The energy density was set as a function of time, with each sample irradiation time controlled by changing the position of the well plate using a two-axis motorized linear stage (SGSP26-150(XY), Sigmakoki, Japan) and a two-axis stage controller (SHOT-702, Sigmakoki). The well plate temperature was maintained at 37°C during light exposure using a microwarm plate (KM-1, As One, Japan) to mimic the human body temperature.

### 2.4 Fluorescence Spectroscopy Analysis

The fluorescence emission from the PpIX solution in Sec. 2.2 was measured after irradiation using a fluorescence microplate reader (Spectra Max Gemini EM, Molecular Devices) at excitation wavelengths of 450 and 505 nm.

### 2.5 Optical Setup for Fluorescence Imaging

**Fig. 2:**
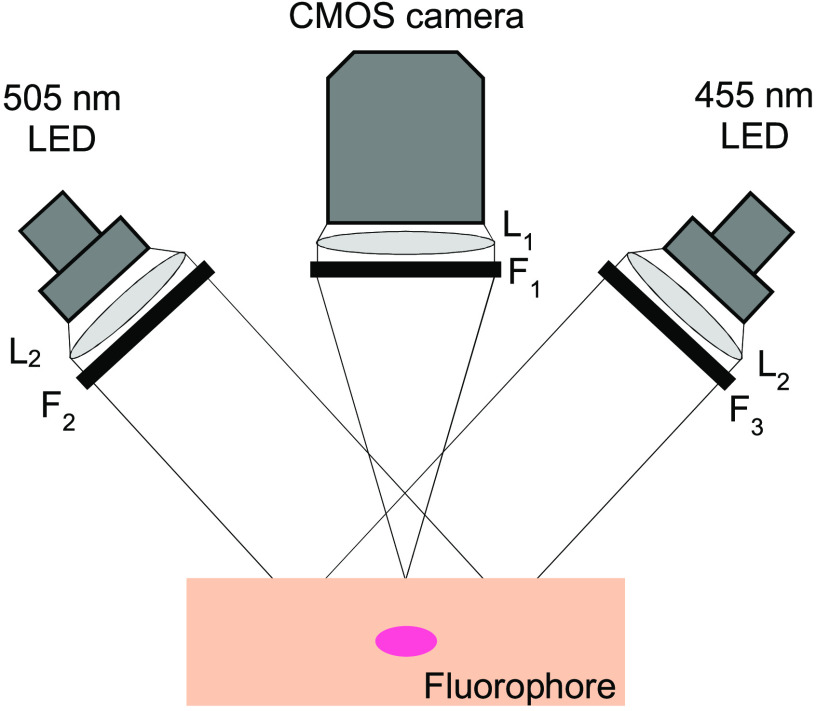
Optical setup for the fluorescence imaging.

Figure 2 shows the setup used in the *ex vivo* and *in vivo* fluorescence imaging of the fluorophore. The fluorescence emission images from the irradiated fluorophore were taken using a CMOS monochrome camera (Chameleon 3, CM3-U3-31S4M, FLIR; with a camera lens $L_{1}$ f=16  mm, F/1.4, NMV-16M1, Navitar), fitted with a 600 nm long-pass filter, $F_{1}$ (FELH0600, Thorlabs) to cut off the excitation light. The exposure time for obtaining the fluorescence images was adjusted between 180 and 5000  μs based on the *ex vivo* or the *in vivo* experiments. The images were obtained for 455 and 505 nm excitations using the 455 and 505 nm LEDs described in Sec. 2.2, with the samples placed on a surface 26 mm from the longpass filter, $F_{1}$. The LEDs used in the excitation were irradiated through a lens, $L_{2}$ (ACL2520U-DG15-A, Thorlabs), and bandpass filters with an FWHM of 25 nm, $F_{2}$ (86-654, Edmund Optics) and $F_{3}$ (86-653, Edmund Optics), for 505 and 455 nm LED, respectively. The LEDs with the bandpass filters, $F_{2}$ and $F_{3}$, resulted in center wavelengths of 500 and 450 nm, respectively. The LEDs were angled 55 deg from the camera and placed approximately equal distances from the sample plate and the camera.

### 2.6 Sample Preparation for Fluorescence Imaging

PpIX (P8293, Sigma-Aldrich) was diluted to a concentration of 1 mM with DMSO (D4540, Sigma-Aldrich). The solution was further diluted to 10  μM with clear epoxy resin (87136, Tamiya, Japan) and was heated in a water bath at 80°C for ∼1  min to deaerate the mixture. The solution was then poured into a petri dish to a height of 1 mm and was left to harden for more than 48 h. PpIX pellets with a diameter of 3 mm were cut out from the hardened epoxy resin sheet. Similar pellets were prepared with the PpIX solution replaced with DMSO. The pellets were irradiated with the green-light LED using the irradiation method described in Sec. 2.3. The power density for the irradiation was $20\;\mathrm{mW}/{\mathrm{cm}}^{2}$ with energy densities of 0, 2, 5, and $10\;J/{\mathrm{cm}}^{2}$. The irradiance and the fluence were chosen to suit the experimental condition, as the initial concentration and the volume of the fluorophore can also affect fluorescence emission intensity and the fluorescence photoswitching of the fluorophore.

### 2.7 Fluorescence Imaging: Pellets and Ex Vivo Analyses

The fluorescence images of the pellets irradiated outside of the mucosa were obtained with the setup in Fig. 2. The fluorescence images were obtained with the LED power density set to $1\;\mathrm{mW}/{\mathrm{cm}}^{2}$ and the camera exposure time of 180  μs. Fluorescence images were also taken from a DMSO pellet for each excitation and were used as a background image.

Porcine stomach purchased from IVTeC Co., Ltd. (Tokyo, Japan) was cut, rinsed in saline to remove mucus, wrapped in aluminum foil, sealed, and frozen at −25°C until use. For the experiment, the porcine mucosa tissue was sliced at an arbitrary depth using a cryotome (CM1850, Leica, Germany), and the PpIX pellet was placed between the upper and the lower slices. The tissue with the pellet was irradiated with the setup in Fig. 2 using the 505 nm LED at a power density of $2\;\mathrm{mW}/{\mathrm{cm}}^{2}$. Fluorescence images were taken after 0, 0.5, 1, 1.5, 2, 2.5, 5, 6, 8, and $10\;J/{\mathrm{cm}}^{2}$ of irradiation for excitations at 455 and 505 nm. The irradiance and the fluence used were chosen to suit the experimental condition, and the parameters used in capturing the images were the same for both excitation wavelengths to allow for a direct comparison.

After obtaining the fluorescence images, the tissue was sectioned to a thickness of 60  μm with the cryotome, and the section was analyzed with a slide scanner (NanoZoomer 2.0 RS, Hamamatsu Photonics, Japan) to determine the mucosal thickness at which the PpIX pellet was placed. The depth was determined by averaging the measured depths at 20 different points.

### 2.8 Mouse Preparation

All procedures involving mice were performed in compliance with the institutional guidelines and regulations and reviewed by the animal experiment and welfare committee of Kochi Medical School.

A seven-week-old female BALB/c nu/nu mouse was housed in a plastic cage with stainless steel grid tops in an air-conditioned room with a 12 h light-dark cycle maintained at room temperature; and it was provided with water and food in the institute for animal experiments of Kochi Medical School.

The human prostate cancer cell line, PC-3, was obtained from Isaiah J. Fidler of the M. D. Anderson Cancer Center, Department of Cell Biology, University of Texas (Houston, Texas). The PC-3 cells were maintained in Dulbecco’s modified Eagle medium (D-MEM: 12100-046, GIBCO) supplemented with 10% fetal bovine serum (Hyclone, SH30910.03, Cytiva, Japan) and kept at 37°C under 5% ${\mathrm{CO}}_{2}$. The PC-3 cells ($2\times 10^{6}$) suspended in 100  μL of D-MEM were injected into the dorsal region at the base of the tail of the mouse subcutaneously. The mouse was used in the experiment after 2 weeks.

A solution of aminolevulinic acid hydrochloride (ALA HCl; Al-05-1, Neopharma, Japan), diluted with distilled water, was injected intraperitoneally at a dose of 600  mg/kg into the tumor-bearing mouse, and the PDD was performed 2 h after ALA HCl administration.

### 2.9 Fluorescence Imaging In Vivo

The skin of the mouse of Sec. 2.8 emitted high fluorescence, which interfered with the fluorescence emission from the lesion. Similar interference has been previously reported.^27^^,^^28^ To specifically observe the fluorescence emitted from the lesion, the skin surrounding the lesion was cut open to expose the lesion, and PDD with 505 nm was performed using the setup in Fig. 2. During lesion exposure to irradiation, the images of the lesion excited at 455 and 505 nm were taken at intervals of $2.4\;J/{\mathrm{cm}}^{2}$ for a total energy density of $9.6\;J/{\mathrm{cm}}^{2}$. The power density of the LEDs used for the PDD and imaging was $∼4\;\mathrm{mW}/{\mathrm{cm}}^{2}$.

## 3 Results

### 3.1 Fluorescence Spectroscopic Analysis

**Fig. 3:**
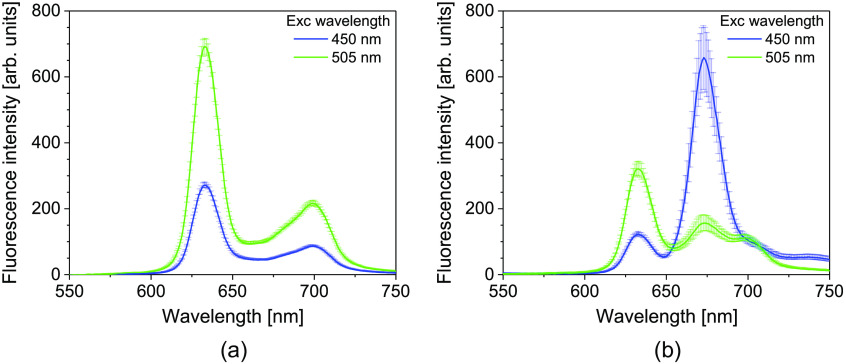
Fluorescence spectra of PpIX (a) unirradiated and (b) irradiated with a 505 nm LED, excited at 450 and 505 nm. The irradiation power density was $20\;\mathrm{mW}/{\mathrm{cm}}^{2}$, and the energy density was $50\;J/{\mathrm{cm}}^{2}$ for the irradiation. The error bars represent standard deviations from three independent experiments.

The fluorescence emission of PpIX solution of Sec. 2.2 before light irradiation, excited with green and blue light, is shown in Fig. 3(a). The fluorescence emission intensity from PpIX excited with blue light and green light is defined as $I_{B}$ and $I_{G}$, respectively. Prior to irradiation, the fluorescence intensity of $I_{G}$ was higher than that of $I_{B}$, with both spectra having their maximum peak at ∼633  nm. After the irradiation [Fig. 3(b)], there was a photodegradation of the 633 nm peak, with an emergence of a peak at around 673 nm. The new peak indicates the formation of a photoproduct. The fluorescence intensity at 633 nm and the intensity at 673 nm were compared before and after light irradiation. The irradiation resulted in a higher $I_{B}$ than $I_{G}$ at the spectra peak of 673 nm. Furthermore, $I_{B}$ at 673 nm was higher than both $I_{G}$ and $I_{B}$ at 633 nm. This indicates the occurrence of fluorescence photoswitching. At this point, the $I_{G}$ at 633 nm reduced to 0.45 times its initial value. In contrast, $I_{B}$ at 673 nm increased to 13.7 times the intensity prior to light irradiation, indicating the accumulation of the photoproduct.

**Fig. 4:**
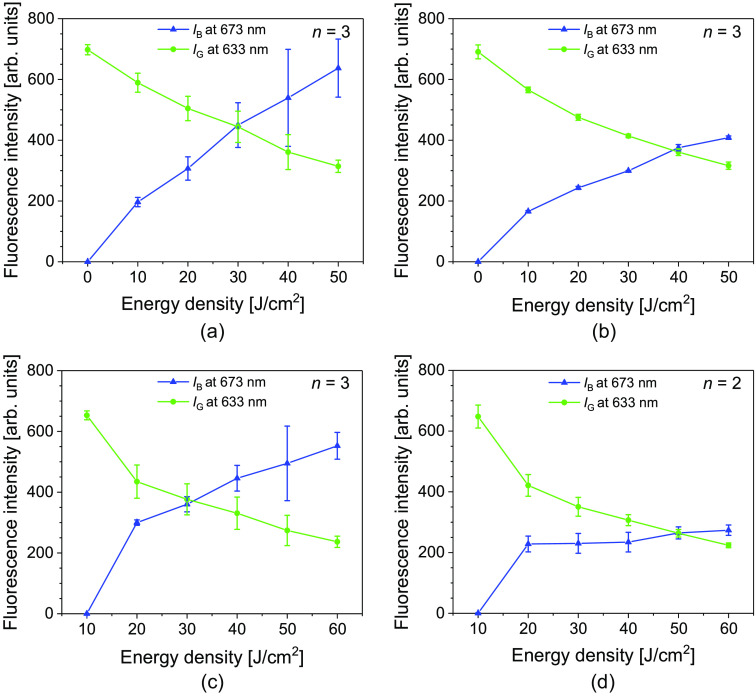
Fluorescence intensity of PpIX exposed to 505 nm, excited at 505 and 450 nm, monitored at 633 and 673 nm at irradiation power densities of (a) 20 and (b) $50\;\mathrm{mW}/{\mathrm{cm}}^{2}$. Fluorescence intensity of PpIX in (a) and (b) further irradiated with blue light with an energy density of $10\;\mathrm{mW}/{\mathrm{cm}}^{2}$, excited at 505 and 450 nm, monitored at 633 and 673 nm at irradiation power densities of (c) 20 and (d) $50\;\mathrm{mW}/{\mathrm{cm}}^{2}$. The fluorescence intensities of $I_{B}$ at 673 nm were corrected to remove the fluorescence intensity of PpIX from that of the photoproduct. The point at which $I_{B}$ and $I_{G}$ cross indicates the point of fluorescence photoswitching. The error bars represent standard deviations.

The changes in the fluorescence intensity with irradiation were monitored at the wavelength peaks of 633 (PpIX) and 673 (photoproduct) nm as the irradiation energy density was increased (Fig. 4). The fluorescence intensity prior to irradiation is assumed to contain only the peaks of PpIX. After irradiation, the spectrum is modified to contain both the peaks of PpIX and the photoproducts. For the analysis of the formation of the photoproduct, the fluorescence intensity at 673 nm was corrected to remove that of PpIX using the method described by Bagdonas et al.^22^ For the two power densities investigated, the pattern of decay of the PpIX fluorescence intensity was similar. On the other hand, the rate of increase of the photoproduct was faster for the lower power density. The fluorescence photoswitching of the fluorescence intensities was observed after the energy densities of 30 and $39\;J/{\mathrm{cm}}^{2}$ for 20 and $50\;\mathrm{mW}/{\mathrm{cm}}^{2}$, respectively.

Further irradiation of each 505 nm irradiated PpIX with 455 nm was performed to determine how the photoproducts react when the excitation light is changed during PDD [Figs. 4(c) and 4(d)]. Irradiation with the blue light led to further PpIX photobleaching for both power densities. Comparing the PpIX photobleaching for each increment of $10\;J/{\mathrm{cm}}^{2}$ between the green and blue light irradiation, the photobleaching was greater for the blue light than for the green light irradiation. Nonetheless, the PpIX photobleaching remained comparable between the two power densities even when the wavelength of the light source was changed. Considerable fluorescence intensity from the photoproduct was maintained after further blue-light irradiation for all energy densities, with each increment in energy density increasing the photoproduct’s fluorescence intensity. However, the fluorescence increase from the blue-light irradiated photoproduct was not as high as in the green-light irradiation.

### 3.2 Fluorescence Imaging in Pellets

**Fig. 5:**
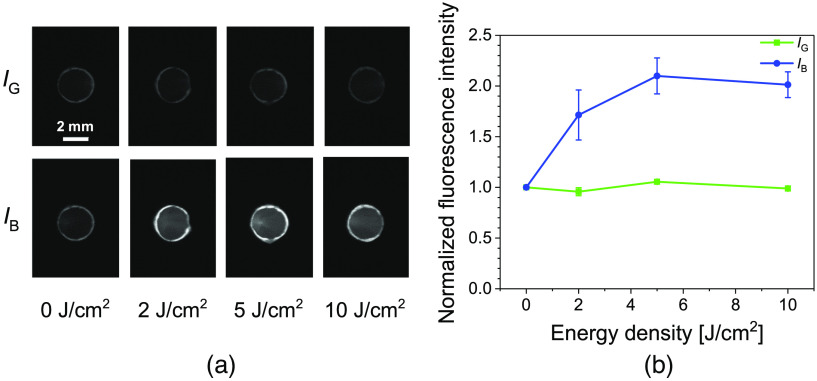
(a) Images of 505 nm irradiated PpIX pellets, and (b) a plot of fluorescence intensity of 505 nm irradiated PpIX pellets with increasing energy density. The error bars represent standard deviations from three independent experiments. $I_{B}$ and $I_{G}$ represent the fluorescence emission intensity from the pellets excited at 455 and 505 nm, respectively.

Figure 5(a) shows the images of 505 nm irradiated pellets. Unlike the spectroscopic analysis, in which the fluorescence intensity of PpIX and the photoproduct can be distinguished to some extent, the irradiation of the pellet excites both PpIX and the photoproduct. The increase in the fluorescence intensity of $I_{B}$ can be observed with irradiation. A spectroscopic analysis of the irradiated pellets showed the emergence of the photoproduct peak at 673 nm. Hence, the increase in the fluorescence intensity with pellet irradiation should result from the formation of photoproducts. Fluorescence photoswitching was observed after $2\;J/{\mathrm{cm}}^{2}$. This was the minimum irradiation time investigated.

The fluorescence intensity values from the pellets were plotted for easier comparison [Fig. 5(b)]. The saturations observed at the edges of the pellets were excluded from the values used in the comparison, and the background intensity was subtracted from the fluorescence images of the PpIX pellets. The unirradiated $I_{G}$ and $I_{B}$ were used to normalize each value. An increase in both $I_{B}$ and $I_{G}$ can be seen after irradiation [Fig. 5(b)], caused by the excitation of both PpIX and the photoproduct at both excitation wavelengths. However, the rise in the intensity of $I_{B}$ was higher than that of $I_{G}$, with the intensity rising to double its initial intensity. For both excitations, the maximum fluorescence intensity occurs at $5\;J/{\mathrm{cm}}^{2}$, after which the photodegradation of the fluorophore is observed. Photoproducts are also susceptible to photobleaching.^21^^,^^24^ Thus, the decrease in the fluorescence intensity of the pellet observed around $10\;J/{\mathrm{cm}}^{2}$ exposure could be caused by both PpIX and photoproduct photobleaching.

### 3.3 Fluorescence Imaging Ex Vivo

**Fig. 6:**
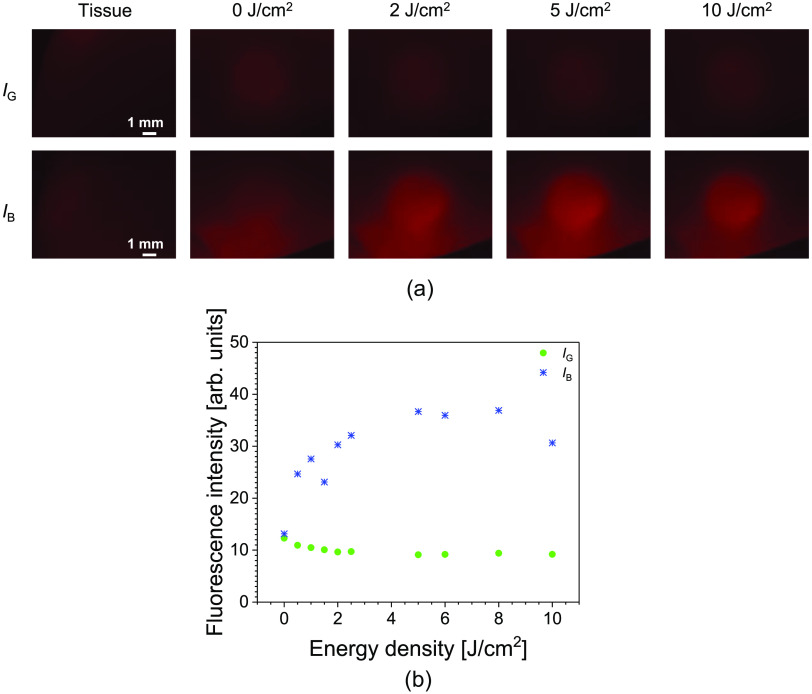
(a) Fluorescence emission images from the fluorophore within the mucosa, with increasing irradiation energy density. (b) A plot of fluorescence intensity against irradiation energy density of fluorophore within the mucosa. The fluorophore was exposed to green light and excited at 455 and 505 nm. $I_{B}$ and $I_{G}$ represent the fluorescence emission intensity from the fluorophore excited with blue light and green light, respectively.

The fluorescence emission images from the fluorophore within the mucosa exposed to green-light are shown in Fig. 6(a). Figure 6(b) shows a plot of the fluorescence intensity of the pellet against the irradiation energy density for each excitation. The mucosa depth at which the fluorophore was placed for this analysis was calculated to be 1.28±0.27  mm from the surface. The tissue fluorescence for both 455 and 505 nm excitations was subtracted accordingly from that of the pellet to account for the background fluorescence effect. The fluorescence emitted from the fluorophore [Fig. 6(a); $0\;J/{\mathrm{cm}}^{2}$] prior to irradiation shows that the intensity was slightly higher for $I_{B}$ than for $I_{G}$. $I_{B}$ was 1.07 times higher than $I_{G}$ after the background fluorescence was subtracted. The increase in $I_{B}$ was observed after $0.5\;J/{\mathrm{cm}}^{2}$ of irradiation. The photodegradation observed in $I_{G}$ was as low as 0.74 times its initial fluorescence intensity, whereas $I_{B}$ increased up to 2.8 times its initial fluorescence intensity. The maximum emission from $I_{B}$ occurred after $8\;J/{\mathrm{cm}}^{2}$. Thereafter, a reduction in the fluorescence intensity was observed. It is difficult to increase the sampling number of this investigation, as cutting the mucosa at the same depth to insert the pellet and having mucosa tissue with similar surface regularity is difficult. Nonetheless, a continual rise in the fluorescence intensity up to $7\;J/{\mathrm{cm}}^{2}$ was observed in another investigation that was performed.

The excitation light sources used in the fluorescence imaging (Fig. 2) have an excitation bandwidth (FWHM) of 25 nm. The additional bandwidth extends the wavelength for the molar excitation coefficient of the fluorophore for each excitation wavelength. Thus, the slightly higher fluorescence intensity of the unirradiated fluorophore seen in $I_{B}$ [Figs. 5(a) and 6(a)] may be attributed to the extended excitation of PpIX. Because the fluorescence comparison is relative to the unirradiated fluorescence intensity for the individual excitation, the extended excitation of PpIX in both $I_{G}$ and $I_{B}$ should pose no issue.

### 3.4 Fluorescence Imaging In Vivo

**Fig. 7:**
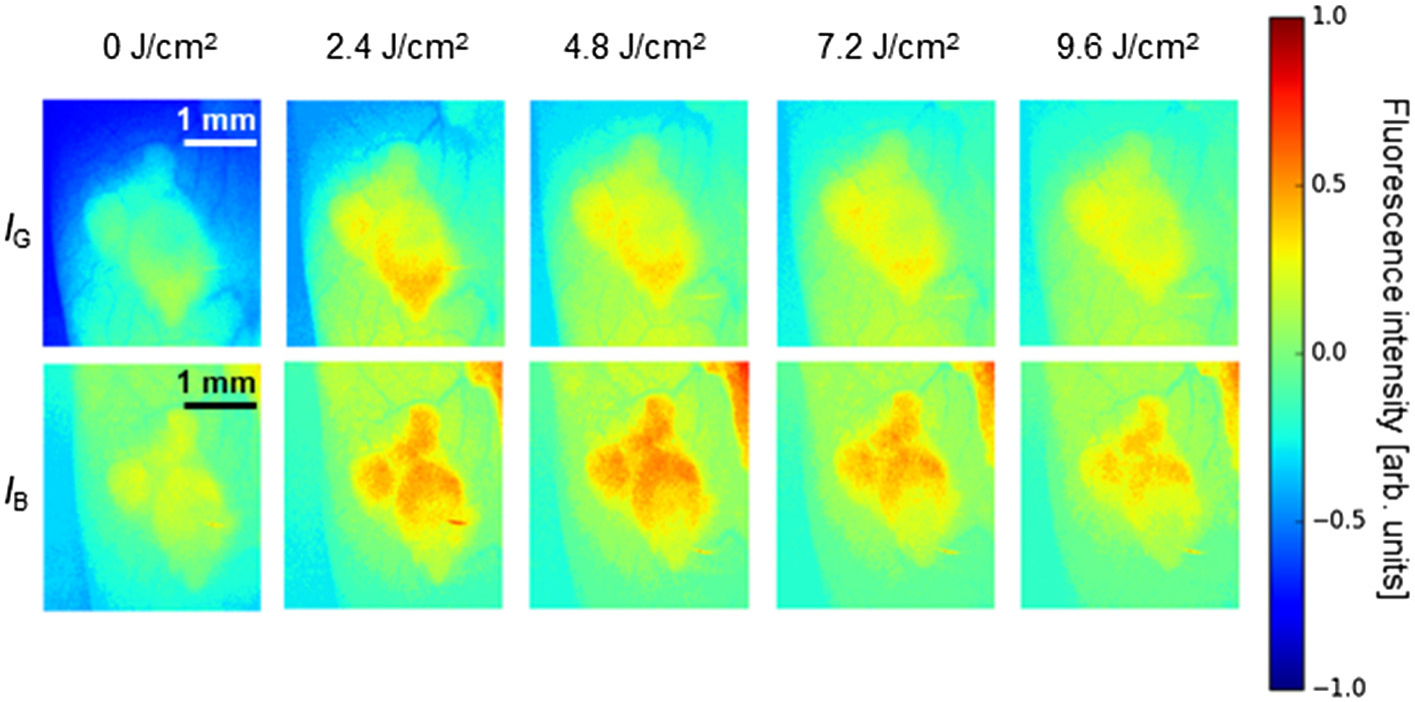
*In vivo* fluorescence emission images of the ALA HCl administered-PC-3 tumor-bearing mouse. The fluorescence images were obtained at time intervals with increased exposure to 505 nm light. At each time interval, images were obtained for green ($I_{G}$) and blue ($I_{B}$) excitation wavelengths.

The fluorescence emission images of the ALA-administered lesion on a mouse with increased exposure to 505 nm excitation are shown in Fig. 7. The background fluorescence emission from the tissue was subtracted from the images. The fluorescence images were normalized using the fluorescence emission at $0\;J/{\mathrm{cm}}^{2}$ exposure for each excitation wavelength and are displayed in a color map for easier visualization of the changes in the fluorescence intensity.

Similar to the *ex vivo* analysis results, the initial fluorescence emission in $I_{B}$ is slightly higher than in $I_{G}$ due to the fluorophore’s extended excitation wavelength to include part of the Q-band of PpIX. This should pose no issue in the result interpretation, considering that the increase in the fluorescence intensity (Fig. 7) is relative to the initial fluorescence emission ($0\;J/{\mathrm{cm}}^{2}$ exposure) for each excitation. For both $I_{B}$ and $I_{G}$, the increase in the fluorescence intensity of the lesion is seen with increased irradiation energy density after $2.4\;J/{\mathrm{cm}}^{2}$. The increase occurs up to $4.8\;J/{\mathrm{cm}}^{2}$, after which the fluorescence intensity begins to decrease. It is worth noting that the continual generation of PpIX from the ALA administered can contribute slightly to the increase in the fluorescence intensity of the lesion.^29^ However, the increase in the fluorescence intensity observed in Fig. 7 is likely caused by the formation of photoproducts. The fluorescence increase should be observed in the same part of the lesion for both blue- and green-light excitations if the increased fluorescence intensity was attributed to PpIX.

## 4 Discussion

The PpIX fluorescence spectrum shape and its maximum peak at 633 nm obtained in our study are consistent with other studies.^20^^–^^22^^,^^24^^,^^30^ The higher initial fluorescence intensity of $I_{G}$ than $I_{B}$ observed in the PpIX solution [Fig. 3(a)] is expected because the absorption band of PpIX is higher at 505 nm than at 450 nm.^11^

The comparison of the PpIX photobleaching rates across the analyses performed for the different PpIX states is difficult because the photophysical property of PpIX is strongly affected by its local environment.^30^ The spectroscopic analysis indicated that the rate at which PpIX photodegraded at the 633 nm peak did not vary with power density for either excitation wavelengths. This result is in line with our previous study of PpIX photobleaching under 405 and 635 nm irradiation.^14^ The consistent photobleaching rate of ALA-induced PpIX over a range of power densities in tumor models and mouse skin *in vivo* has also been reported.^31^^,^^32^ On the other hand, a discrepancy was seen in the findings reported by others, in which PpIX photobleaching was dependent on the power density.^18^^,^^33^ These studies claimed that the variation in the photobleaching rate was due to oxygen availability in the target area. In the results of Iinuma et al.^31^ and Sørensen et al.,^32^ in which no dependence on the power density was observed, no oxygen effect was reported in the region of interest for the fluence rate analyzed. A similar condition may be assumed in our solution, pellet, and *ex vivo* analysis, as it does not involve a vascular supply of oxygen. In addition, the photobleaching mechanism could be oxygen-dependent or oxygen-independent.^30^^–^^32^ Thus, the observed photobleaching mechanism in our study could also be oxygen-independent. In our study, the PpIX photobleached with a half-life at $40\;J/{\mathrm{cm}}^{2}$, which is within the previously reported range.^17^

The fluorescence emission from the pellets [Figs. 5(b) and 6(b)] were analyzed based on fluorescence emission images and included the fluorescence intensity of simultaneously excited PpIX and the photoproducts. Hence, analyzing the fluorescence photobleaching rate of PpIX from the fluorescence images would not be accurate. A spectroscopic analysis of the irradiated PpIX pellets performed across various power densities showed comparable photobleaching rates at the 633 nm spectra peak. This further confirms the independence of the PpIX photobleaching rate with the fluence rate.

The photoproduct observed in our experiment has its peak at 673 nm, confirming that the photoproduct formed in our analysis is the chlorin-type photoproduct, Ppp.^22^^,^^24^ The peak of Ppp is detected for both 450 and 505 nm excitation wavelengths. As previously reported, a high Ppp emission intensity relative to that of PpIX was observed for excitation at 450 nm (Fig. 3).^22^ Increased fluorescence intensity of Ppp is seen for PpIX irradiation at lower power densities (Fig. 4). In line with our observation, a more intense photoproduct absorption from irradiated hematoporphyrin at a low irradiation dose was reported by others.^34^ The reason for the varied formation rate of Ppp with the power density is not certain. The formation of the chlorin-type photoproduct is dependent on factors such as illumination time, the initial concentration of the photosensitizer, oxygen, and the pH of the surrounding.^30^^,^^34^ Ppp is primarily formed by the reaction of PpIX with singlet oxygen. The reaction rate between these two may vary based on the fluence rate. In addition, oxygen availability may play a role in the Ppp formation rate.^30^

The above comparisons corroborate that the results obtained in this study are in line with reports of other studies. Thus, the methods of analysis applied in this study can be verified.

The experimental results show that the combined use of fluorescence emission from PpIX and Ppp can possibly increase the fluorescence emission observed from deeply located tumors. With fluorescence photoswitching, a fluorescence intensity similar to or even higher than the initial fluorescence emission from the PpIX can be obtained.

The fluorescence photoswitching time, the fluorescence emission intensity from blue-light excitation relative to the green-light excitation at fluorescence photoswitching ($I_{B}/I_{G}$), the maximum fluorescence emitted from the blue-light excitation ($I_{B\;\mathrm{Max}}$), and the time it takes to attain it for each analysis are summarized in Table 1. The values in the table were obtained based on the discrete irradiation points analyzed in this study. Fluorescence photoswitching in pellets, *ex vivo*, and *in vivo* occurred at the first analysis point. Thus, the fluorescence photoswitching time should be less in these cases.

**Table 1:** Summary of fluorescence photoswitching time and relative fluorescence intensity obtained for each analysis.

	In solution ($20\;\mathrm{mW}/{\mathrm{cm}}^{2}$)	Pellet	*Ex vivo*	*In vivo*
Fluorescence photoswitching time (s)	1500	100	500	600
$I_{B}/I_{G}$ at fluorescence photoswitching (−)	1.01 ± 0.08	2.76 ± 0.71	2.25	1.47
$I_{B\;\mathrm{Max}}$ time (s)	2500	250	8000	1200
$I_{B}/I_{G}$ at maximum (−)	2.05 ± 0.17	3.04 ± 0.55	3.92	1.65
$I_{B\;\mathrm{Max}}/I_{G\text{ initial}}$ (−)	0.93 ± 0.11	3.21 ± 0.58	3.00	0.89

The standard deviations are obtained from three independent analyses.

The amount of PpIX used in the spectroscopic analysis is at least double that used in *ex vivo* analyses. Thus, the observed time for fluorescence photoswitching is longer in the solution analysis than in the *ex vivo* analysis. Based on the trend in Fig. 4(c), changing the irradiation light source to blue allows for maintaining a higher $I_{B}$ than $I_{G}$ for at least 18 min. Because no photobleaching of $I_{B}$ was yet observed within the investigated irradiation time, a further rise in the $I_{B}$ with further irradiation may be possible, and the fluorescence observation time for higher $I_{B}$ than $I_{G}$ may be longer. The attenuation and scattering of the irradiation light and the fluorescence emitted from the pellet may have caused the longer irradiation time before fluorescence photoswitching in the *ex vivo* experiment compared with the pellet experiment outside the tissue. The fluorescence photoswitching *in vivo* was also observed at the minimum irradiation. Unlike the solution and *ex vivo* analysis, in which the fluorescence photoswitching is primarily dependent on the fluence rate, other factors such as the concentration of ALA converted to PpIX during imaging, the distribution of PpIX within the tumor, and the oxygen availability at the site of diagnosis have a key effect on the fluorescence photoswitching. Thus, a variation in the fluorescence photoswitching time and the intensity of $I_{B}$ is expected, and optimization is necessary during actual PDD.

For ALA-PDD, the benefit of 505 nm excitation to red light excitation for deeply located tumors was confirmed by Ihara et al.^11^ They reported that PDD with 505 nm extended the diagnostics depth for lesions located at a depth of 1.1 mm or more in the mucosa. However, our results show that PpIX photobleaching also occurs with 505 nm irradiation and excitation. Thus, although at the initial state of PpIX excitation, 505 nm is suitable for detecting deep intramucosal tumors, a reduction in the fluorescence emission from PpIX during fluorescence detection is expected.

Our *ex vivo* analysis was performed in porcine gastric mucosa at a depth of about 1.28 mm. This depth is within the depth suitable for the green light PDD reported.^11^ The similarity between the light penetration depth in the human gastric mucosa and porcine gastric mucosa has been confirmed.^11^^,^^35^ Our *ex vivo* investigation was performed using a similar porcine gastric mucosa as Ihara et al.^11^ With that stated, it is safe to follow the clinical applications reported in their study. Our result shows that the successive use of 505 nm followed by 450 nm excitation is advantageous for the ALA-PDD of deeply located lesions. ALA-PDD with fluorescence photoswitching may be applied in detecting undifferentiated adenocarcinoma in gastric and other mucosal tissues with similar absorption and scattering coefficients to the gastric mucosa. In addition, because photoproducts are also formed from other forms of porphyrin photosensitizers,^34^^,^^36^^,^^37^ the diagnostics efficacy of PDD with other photosensitizers may be improved with fluorescence photoswitching.

The Ppp formation primarily depends on the concentration of the photosensitizer and the oxygen available at the tumor location.^22^^,^^30^^,^^38^ Based on our result, it is also dependent on the power density of the irradiation light, i.e., the irradiation time. In addition, the fluorescence emission intensity of Ppp is dependent on the excitation wavelength.^17^^,^^22^ The variation in the photoproducts formed based on these factors may make photoswitching advantageous for PDD and PDT monitoring. Observing the Ppp formation by 450 nm excitation during diagnostic and treatment can be used to estimate how much fluence was actually used during PDD and PDT.

The photosensitizer and the oxygen concentrations are difficult to control *in vivo*. PpIX is continuously generated from ALA, the generated amount of PpIX is not fixed, and PpIX relocalizes during PDD.^29^ Thus, optimization for the fluorescence photoswitching condition is primarily based on the light source. As stated earlier, the Ppp formation is higher for lower power densities, likely due to slower oxygen depletion and the reaction rates between PpIX and oxygen. Because is difficult to control these factors, a lower power density is recommended for fluorescence photoswitching in PDD. It is also necessary to bear in mind that Ppp photobleaches and the rate may depend on the power density.

The formation of Ppp *in vivo* may be speculated by the increase in the fluorescence intensity with light irradiation. The potential of fluorescence photoswitching was also demonstrated. However, the lesion in the *in vivo* analysis was ∼167.6  μm from the surface. In addition, the lesion was exposed to the irradiation light by peeling back the skin surrounding the lesion during light exposure. Thus, *in vivo* analysis for lesions located deeper is needed to verify fluorescence photoswitching in deeply located tumors.

Although the decrease in the formation of Ppp with a higher fluence rate was confirmed in the spectroscopic and the *ex vivo* analysis, this could not be verified in the *in vivo* analysis due to the limited power output of our optical setup. The power densities used in some clinical PDD with 405 nm are lower than those used in this study.^39^^–^^41^ Nonetheless, our investigation shows that photobleaching rates are comparable between power densities. Thus, the effect of photobleaching with lower irradiance is expected to be similar to that of higher irradiance. We also found that Ppp formation increases with lower power density. Hence, the use of lower power densities in PDD further improves tumor visualization. In other cases, a much higher fluence rate may be used,^1^^,^^3^^,^^4^^,^^42^ making investigation of the effect of the higher fluence on the Ppp formation *in vivo* necessary. In addition, an endoscopic analysis would also be useful in demonstrating the potential of fluorescence photoswitching.

## 5 Conclusion

We have investigated fluorescence photoswitching by observing the fluorescence emission from the primary excitation of PpIX followed by the primary excitation of Ppp using 505 and 450 or 455 nm excitations, respectively. The phenomenon was observed in all states of PpIX investigated in this study. The penetration depth of 450 nm is known to be less than that of 505 nm. However, our *ex vivo* experimental result showed that the fluorescence intensity emitted from 450 nm excitation of the fluorophore was higher than that from 505 nm excitation at a depth of about 1.28 mm. This depth is within the previously reported depth suitable for the green light PDD of deeply located tumors. Although the increase in the fluorescence observation is dependent on the fluence rate of the light source and other factors, the potential of fluorescence photoswitching for increasing the fluorescence observation intensity of deeply located tumors has been demonstrated. It is expected that this method can be applied to other types of cancer located in tissue having similar tissue optical properties as that of gastric tissue. In addition, there is a potential to extend PDD with fluorescence photoswitching to other photosensitizers that generate photoproducts upon irradiation.
