# Supplementary material for: Increased fluorescence observation intensity during the photodynamic diagnosis of deeply located tumors by fluorescence photoswitching of protoporphyrin IX
Source: J Biomed Opt. 2023 May 15;28(5):055001. doi: 10.1117/1.JBO.28.5.055001 (PMC10185104; doi:10.1117/1.JBO.28.5.055001)
Supplement: Supplementary file 1 [file JBO_028_055001_SD001.pdf]

Increased fluorescence observation intensity during the photodynamic diagnosis of deeply located tumors by fluorescence photoswitching of protoporphyrin IX — Supplementary Material

Sochi J. Ogbonna,[a,*] William Y. York,[a,b] Takahiro Nishimura,[a,] Hisanao Hazama,[a] Hideo Fukuhara,[c] Keiji Inoue,[c] and Kunio Awazu[a,d]**
[a]Osaka University, Graduate School of Engineering, Division of Sustainable Energy and Environmental Engineering, Osaka, Japan
[b]National Institute on Aging, Laboratory of Clinical Investigation, Baltimore, MD, USA
[c]Kochi University, Kochi Medical School, Department of Urology, Kochi, Japan
[d]Osaka University, Global Center for Medical Engineering and Informatics, Osaka, Japan

S1 Absorption Spectra of Irradiated Protoporphyrin IX

Figure S1 shows the absorption spectra of PpIX before and after 50 J/cm^2 irradiation with a 505 nm light set at an irradiation power density of 20 mW/cm^2. The irradiated absorption spectrum of PpIX is thought to include the peaks of the PpIX photoproduct, photoprotoporphyrrin (Ppp). Similar to other studies, there is the photobleaching of the PpIX peaks, the broadening of the Soret band, and the emergence of a peak at 667 nm with light irradiation. [22,23] A ratio of the irradiated absorption spectra to the unirradiated absorption spectra showed peaks at 520 nm in addition to the 450, 560, and 615 nm observed by other studies.[22]

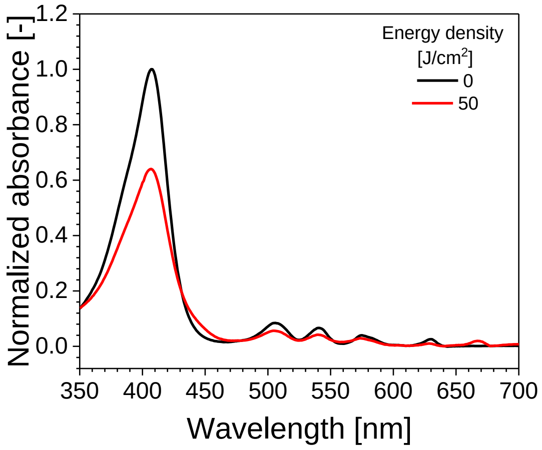

Fig. S1 Absorption spectra of PpIX (a) unirradiated and (b) irradiated with a 505 nm LED. The irradiation power density was 20 mW/cm^2, and the energy density was 50 J/cm^2 for the irradiation. The spectra have been normalized using the intensity at 408 nm before irradiation.
